# Synchrony in Broadband Fluctuation and the 2008 Financial Crisis

**DOI:** 10.1371/journal.pone.0077254

**Published:** 2013-10-28

**Authors:** Der Chyan Lin

**Affiliations:** Department of Mechanical and Industrial Engineering, Ryerson University, Toronto, Ontario, Canada; University of Catania, Italy

## Abstract

We propose phase-like characteristics in scale-free broadband processes and consider fluctuation synchrony based on the temporal signature of significant amplitude fluctuation. Using wavelet transform, successful captures of similar fluctuation pattern between such broadband processes are demonstrated. The application to the financial data leading to the 2008 financial crisis reveals the transition towards a qualitatively different dynamical regime with many equity price in fluctuation synchrony. Further analysis suggests an underlying scale free “price fluctuation network” with large clustering coefficient.

## Introduction

Synchrony among coupled oscillators has fascinated scientists and engineers for decades. There is an extensive literature on the subject, from Huygen's clock [1], amplitude, phase synchronization of low-dimensional systems [2], Kumamoto phase oscillators [3], to diverse manifestations in natural phenomena, such as social opinion formation [4]–[7], population of species [8], [9], collective motion of Starling flock [10], traffic [11], neuronal dynamics [12], [13], just to name a few. Synchrony between broadband processes is more subtle for it can emerge, disappear and reappear sporadically in time, and is difficult to identify for it is less exact; i.e., only similar fluctuation pattern may exist. Of interest here is the synchrony between broadband processes showing scale free characteristics. The fluctuation of the process has therefore a power law characterization, such as the power law power spectrum. In this work, we propose the state of *fluctuation in synchrony* [14] (FIS) between broadband processes and present a wavelet method to derive phase-like quantity to study FIS.

One area where FIS likely occurs is the market dynamics. Equity price is known to exhibit broadband fluctuation and fractal property [15], [16]. Besides the market fundamental, a broad base price increase or drop suggests the possibility of FIS. The goal of this work is to study FIS of the dramatic equity price fluctuation in the 2008 financial crisis. We find that the FIS among price series exhibits a bifurcation pattern at the crisis. Using a network analogy, we further show that the market dynamics underlying FIS has a scale free configuration and a large clustering coefficient.

### Methods

**Figure 1:**
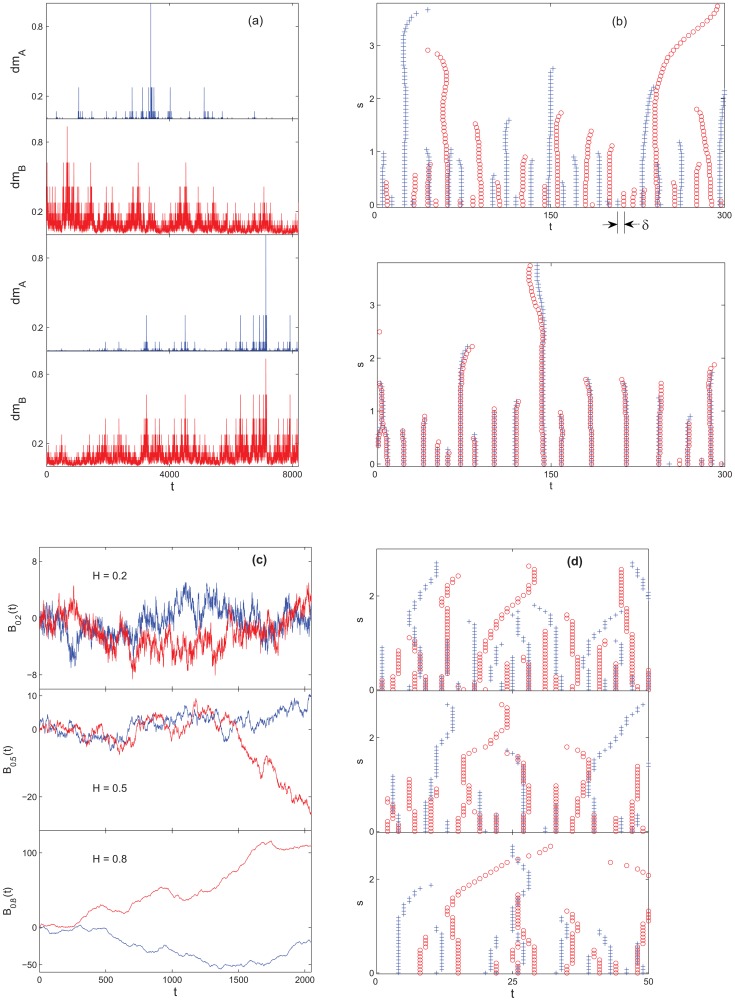
FIS in artificial examples. (a) Cascade 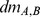 for 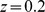 (top two panels) and 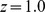 (bottom two panels). Notice the more (synchronized) similar fluctuation pattern as 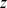 increases to 1. (b) WTMML's of 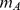 (“+”), 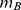 (“o”) constructed from (a) with 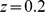 (top) and 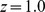 (bottom). (c) Two samples (one in blue and one in red) of 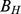 for 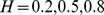 (top to bottom). (d) WTMML's of 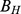
's shown in (c): (“+” for the blue sample and “o” for the red sample).

Our approach relies on the so-called wavelet transform modulus maxima line (WTMML). For square-integrable function or bounded signal in practice, wavelet transform can effectively describe the local fluctuation according to the scale of resolution [17], [18]. The use of wavelet transform as a signal processing tool to describe scale free property has a long history [17]–[19]. WTMM was initially motivated for improved understandings of the hydrodynamic turbulence, but was soon realized to have much wider application for natural processes in diverse fields. The main idea is based on the concept of singularity in mathematics where scale free property can be obtained from the wavelet coefficients along the WTMML. Our objective is slightly different in that we are mainly interested in the location of the WTMML. On the wavelet time-scale plane 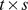, WTMML is a connected curve defined by the modulus maxima of the continuous wavelet transform coefficient 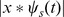, where 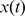 is the time series of interest, a “
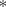
” denotes the convolution, and 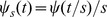 is the dilated analyzing wavelet 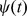 satisfying 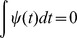 [17]. We shall denote these WTMML locations at the smallest scale by 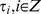, and refer to them as the *WTMML roots*. Huang and Mallet proved the seminal result on the convergence of 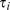
's towards (singularity) locations where the derivative of the function is undefined [17]; see also Ref [19]. Intuitively, these are where the time series exhibits abrupt changes or jumps in the amplitude fluctuation. By identifying such feature as the phase setting event, 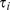 maybe used to derive the phase difference of the fluctuation of broadband processes. Specifically, let 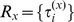, 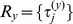 be the WTMML roots of time series 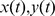, respectively. The first step in the FIS analysis is to search for nearest WTMML roots, say 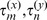, satisfying 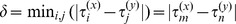 (FIG. 1b below). Once such nearest WTMML roots are found, they are deleted from 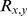 and the search continues until all WTMML roots are processed. The 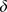 so obtained captures the difference in “timing” of the large amplitude jumps of 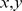, and is used as the measure for the phase difference of the fluctuation.

When all WTMML roots of 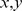 are separated by a fixed distance, 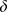 equals a constant and has a Dirac delta probability density function (PDF) 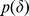. In this case, the FIS is said to be *complete*. As the FIS level drops, the WTMML roots are more scattered and the shape of 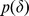 widens. To measure FIS, we use an entropy based synchronization index (SI) [20]: 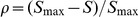 where 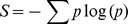 is the Shannon entropy and 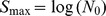 is the largest entropy from a uniformly distributed 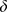. Thus, 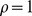 for a Dirac delta 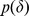 in complete FIS and 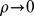 for completely independent fluctuations.

In the numerical analysis, the 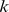
th order gaussian derivative wavelet 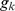 is considered. Since 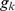 has 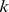 vanishing moments, it is able to capture irregularities in the 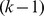
th derivative of a function. However, for defining the phase event, wavelet with higher order vanishing moment may be too sensitive, resulting in WTMML roots at every “regular” rise-and-fall of the time series. To capture more “violent” fluctuation pattern that is typically found in the financial data, using analyzing wavelets with the lowest number of vanishing moment is more desirable. In this study, 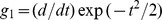 is used.

## Results

We will first apply the above ideas to demonstrate FIS using artificial time series. Consider multifractal measures 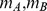, where 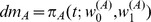, 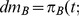 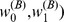, are dyadic cascades with deterministic weights 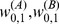 satisfying 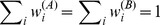. Let the weights be assigned randomly, say, by flipping a coin, to dyadic intervals. One can write 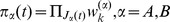, where 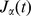 is a random sequence of 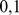. FIS is realized by ensuring 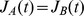 in some time interval. This may be achieved by imposing 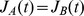 whenever a uniformly distributed 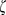 in [0,1] is less than a constant 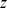. Thus, 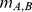 are independent when 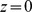 and in complete FIS when 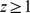. The function 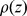 is therefore increasing in 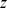. To demonstrate, 30 pairs of 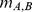 of 8,192 points each were generated with 
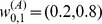
, 
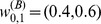
 for 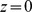 to 1 in 0.2 increment. Indeed, it is observed that the WTMML roots are more scattered at 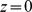 and have almost identical locations at 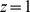 (FIGs. 1a, 1b). They lead to the increasing 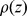 as predicted (TABLE 1).

**Table 1:** Average 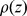 with 1 standard deviation (SD) based on the ensemble of 30 pairs of 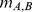.

g	0.0	0.2	0.4	0.6	0.8	1.0
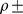 SD	0.661 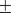 0.003	0.674 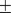 0.004	0.695 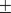 0.005	0.732 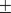 0.005	0.797 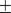 0.006	0.952 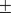 0.032

While 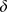 is motivated to measure the phase difference, it also characterizes the intrinsic property of the broadband fluctuation. Consider the fractional Brownian motion (fBm) 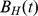 [21]. With probability 1, 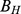 is continuous and nowhere differentiable; i.e., it is singular almost everywhere (Lipschitz exponent less than 1). Its covariance function given by 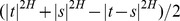 implies a negatively (positively) correlated increment 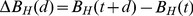 for 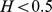 (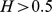). *For a fixed* 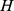, we conducted the FIS analysis based on different realizations of 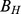 and found a decreasing 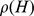; see FIGs. 1c, 1d and TABLE 2). This result follows intuitively from 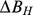. Since the singularity in 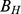 arises mostly from a positive increment followed by a negative one, or vice versa, there are relatively more WTMML roots for 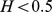 (with negatively correlated increment). This implies a smaller 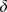 range, or narrower 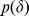, and thus a larger 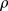 value.

**Table 2:** Average 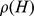 with 1 standard deviation (SD) based on 30 samples of 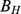 at 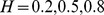.

H	0.2	0.5	0.8
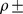 SD	0.581 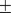 0.009	0.506 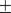 0.012	0.423 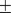 0.011

**Figure 2:**
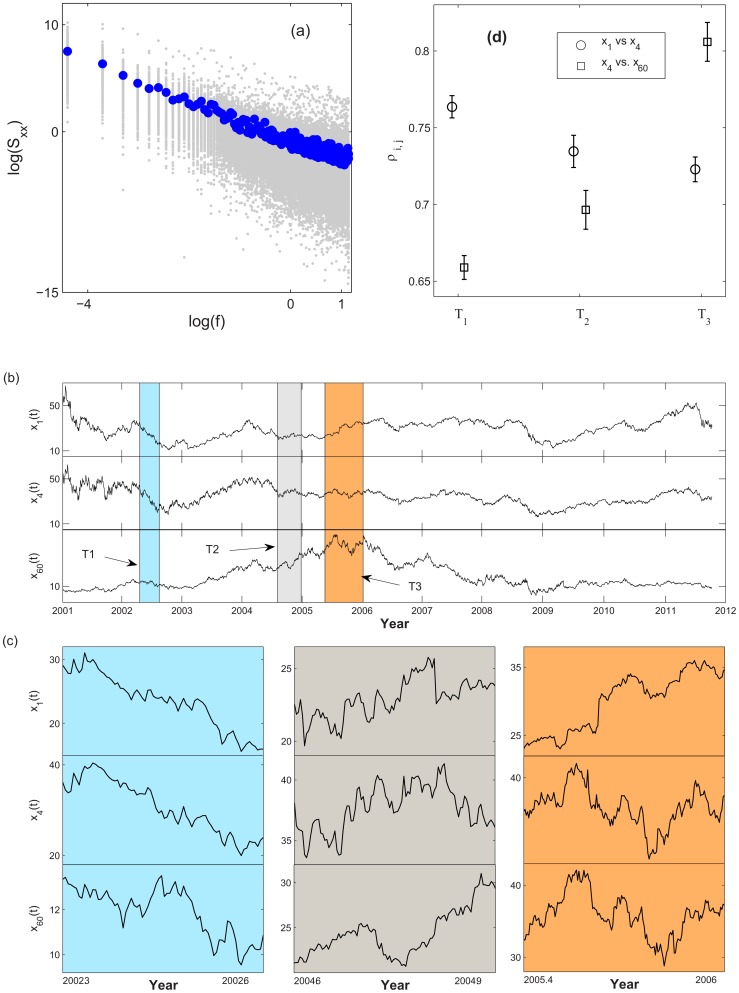
FIS in equity price series. (a) Power law power spectra of all price series (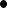, grey) and their average (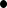, blue), (b) Examples of price series 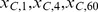 (top to bottom). Typical FIS is demonstrated in three selected time intervals: 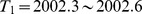 Yr (left, blue background), 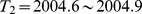 Yr (middle, grey background), 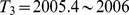 Yr (right, orange background). (c) Zoom-in of the price fluctuation of the price series in 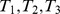 (left to right). (d) Pair-wise 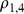 (“o”), 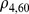 (“
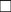
”) in 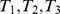 (left to right). Calculations are based on 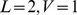. Error bars correspond to one standard deviation of the 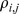 values in 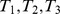.

We now apply these ideas to analyze the fluctuation in the equity price series. The daily closing price, 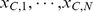, for 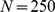 publicly traded stocks in NASDAQ from Jan 2001 to Nov 2011 are used (File S1). This selection is aimed at a reasonable mix of the market, so that any potential FIS represents a general market property, rather than the characteristics of a particular business sector. FIG. 2a shows the broadband character of the price series where the power-law power spectrum can be clearly seen. FIGs. 2b 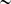
2d shows the typical case that suggests FIS in the market dynamics. They are the closing price of two technology companies 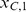 (Agilent Tech Inc) and 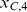 (Analog Devices Inc), where similar fluctuation pattern is visually apparent. But similar fluctuation patterns exist more generally in companies from different sectors, for example, those between 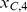 and 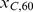 of an energy company (CenterPoint Energy Inc).

To analyze the potential FIS in the price series, we take into account the market nonstationarity by processing the data in window segments of 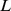 calendar year, advancing every 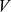 calendar month (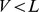). In general, using smaller 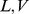 suffers from poorer statistics and results in larger fluctuation of the SI 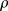. But using larger 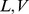 can average out the subtle fluctuation in the market dynamics. Our goal is to find persistent FIS indicators over a reasonable range of 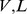. These indicators are now described.

Given 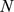 price series, there are 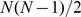 sets of 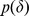
's are obtained in each of the window segment (31,125 sets for 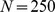). They were then used to calculate the pair-wise SI 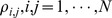. The FIS indicators used in this study are defined from the 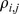. First, the sample mean and standard variance 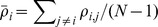, 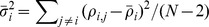, respectively, are calculated for the 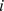
th price series. Then, averages are made to define the FIS indicators 
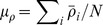
 and 
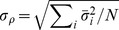
.

A large 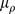 implies a higher FIS level market. But it does not mean the formation of a global cluster of companies that exhibit similar price fluctuation. If 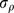 is also large, smaller clusters are likely formed. This is because, while 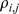 for 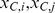 in the same cluster increases, the 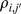 with the price series 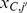 from a different cluster decreases, which in turn creates a greater disparity of the pair-wise SI and, thus, a larger 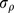.

**Figure 3:**
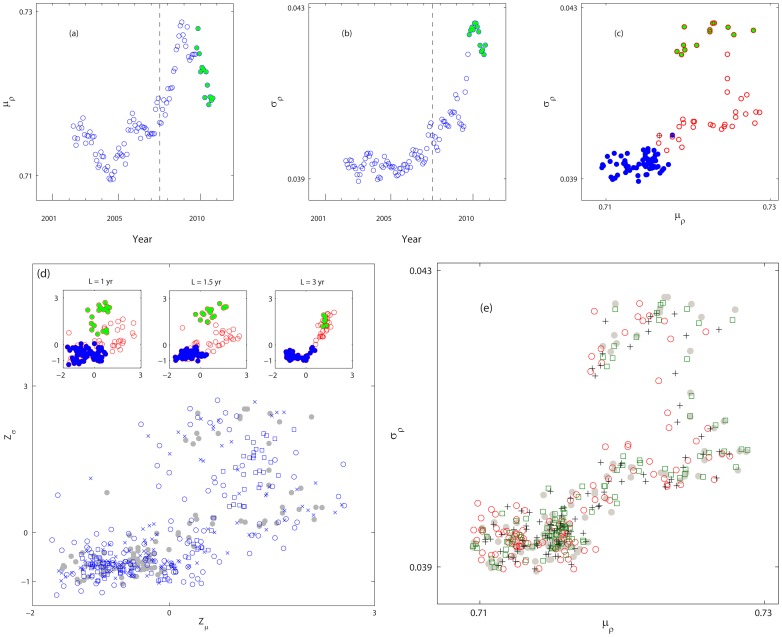
FIS indicators of the price series obtained by using window parameters 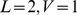 from 2001 to 2011. (a) 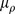, (b) 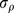, (c) 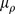 vs. 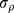. The transition year 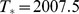 is marked by the long-dashed line in (a), (b) and by a cross 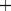 in (c). In (c), blue filled circles, and red open circles correspond to the pre- and post-2008 regimes. In (a)
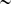
(c), green filled circles correspond to the top branch of the bifurcation pattern. (d) FIS indicators obtained by using different window lengths 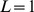 (“o”), 1.5 (“
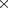
”), 2 (“
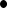
”, grey) and 3 (“
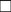
”) and 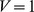. To facilitate the comparison, 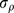 vs. 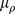 are normalized according to 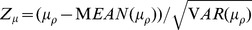 and 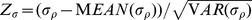 where MEAN, VAR denote the sample mean and variance, respectively. The insets show the transition separating the pre- and post-2008 regimes using the same color scheme as (c). (e) 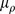 vs. 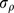 for the first 100 stocks (“o”, red), 150 (“
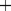
”, black), 200 (“
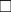
”, green). The result of 250 stocks shown in (c) is added for comparison (“
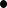
”, grey).

FIGs. 3a, 3b present the FIS indicators that characterize the market synchrony. They show the 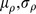 obtained by using window parameters 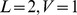. Most notably here is the rise of 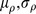 at apparently a *transition year* 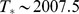 Yr. Let 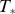 define a pre- and post-2008 regimes in reference to the 2008 financial crisis. A closer examination reveals a bifurcation pattern, where 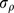 considered as a function of 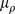 first clusters around a steady range in the pre-2008 regime and then bifurcates into at least two branches in the post-2008 regime (FIG 3c). The rising 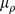 and 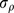 after 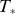 suggest the formation of smaller clusters of 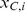
's showing similar price fluctuation. The top branch in the post-2008 regime started at around the third quarter of 2009. It is when 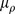 begins to drop, showing the “return” of the market to its pre-2008 FIS level, while 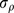 remains at a higher post-2008 level, showing the persistence of the market with a smaller cluster configuration.

These FIS characteristics, the market transition at 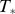 and the bifurcation pattern, are also captured by using different window parameters 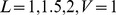 (FIG. 3d), and using different number of price series (FIG. 3e). The robustness against these technical parameters supports a genuine market phenomenon in the analyzing period. For 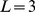, however, these characteristics are no longer found. Note, the “return” of the 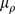 value since the second half of 2009 (FIG. 3c) suggests a 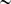
2 years “lifespan” for these FIS features. As a result, the missing of these characteristics using the 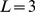 window segments should be due to averaging. In what follows, we will report results obtained by 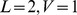.

It is reasonable to assume the action from traders has an immediate impact on the observed FIS in the price series. To examine this potential link, we also consider FIS from the daily returns. In particular, let 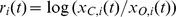 be the return during regular trading hours (9:30 am
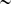
4:00 pm) where 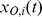 is the opening price of the 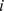
th stock on the 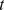
th trading day. Consider the cumulative daily return up till the trading day 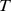, 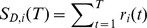, and the cumulative after-hour return (4:00 pm
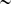
8:00 pm, 7:00 am
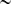
9:30 am, E.T.), 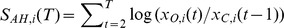. Note that subtle differences have been noted in the after-hour trading such as its lower liquidity, larger bid-ask spread [22]. Note, also, that 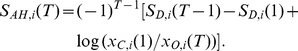
(1)


Hence, in terms of the fluctuation pattern, the after-hour 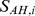 may be viewed as a perturbation of the 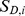 by the opening price 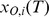. As a result, these returns can exhibit qualitatively different FIS characteristics.

**Figure 4:**
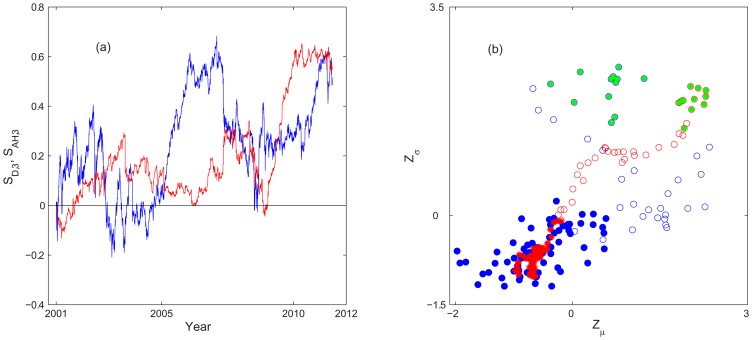
FIS indicators of the cumulative returns. (a) Examples of cumulative daily and after-hour returns for a pharmaceutical services company (AmerisourceBergen Corp), 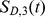 (blue) and 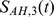 (red). (b) FIS indicators of 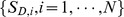 (blue) and 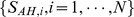 (red). The indicators are normalized: 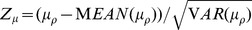 and 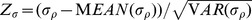. The pre-, post-2008 regimes are shown using the similar specification as FIG. 3c: pre-2008 regime in filled circles, post-2008 regime in open circles, and solid green circles from the period of the top branch in FIG. 3c.

Similar to the price series, our result shows the same ascending trend in 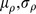 of the 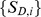, 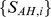, indicating a more synchronized trading action in the crisis development (FIG. 4). FIG. 4b shows the FIS indicators of 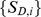 where similar FIS characteristics reported in FIG. 3 are observed. However, they are not found in the FIS indicators of 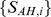. These results suggest a more direct impact to the market FIS from the regular-hour trading activities. That being the case, it is not possible to reject the significance of the after-hour trading in its entirety, since different trading characteristics in the pre-open (7:00 am
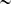
9:30 am) and post-close (4:00 pm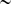 8:00 pm) are believed to exist [22]. However, we are not able to analyze these periods separately in the after-hour trading in this work.

**Figure 5:**
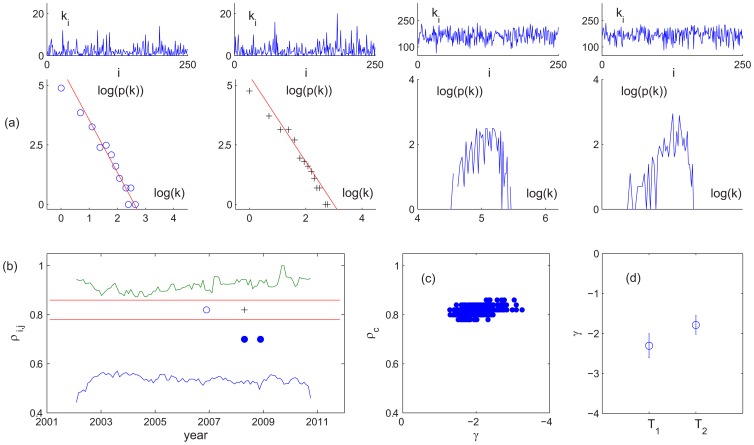
Network characterization of FIS: degree of the network. (a) Degree 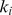 and degree PDF of the price series, from left to right: 2007 Yr (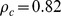, “o”), 2008 Yr (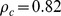, “+”), and 2008 Yr, 2009 Yr (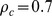, “
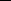
”). The solid lines shown in the two left panels have the slope −2.2 and −1.8, respectively. (b) The boundaries of the maximum (green) and minimum (blue) of 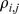 of the price series. The horizontal lines show the range of 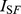 where 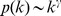. The particular cases shown in (a) are also marked. (c) The exponent 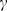 determined for 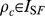 (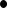) where 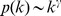 is observed. (d) Average 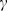 in the pre- and post-2008 regimes. The error bars correspond to one standard deviation.

**Figure 6:**
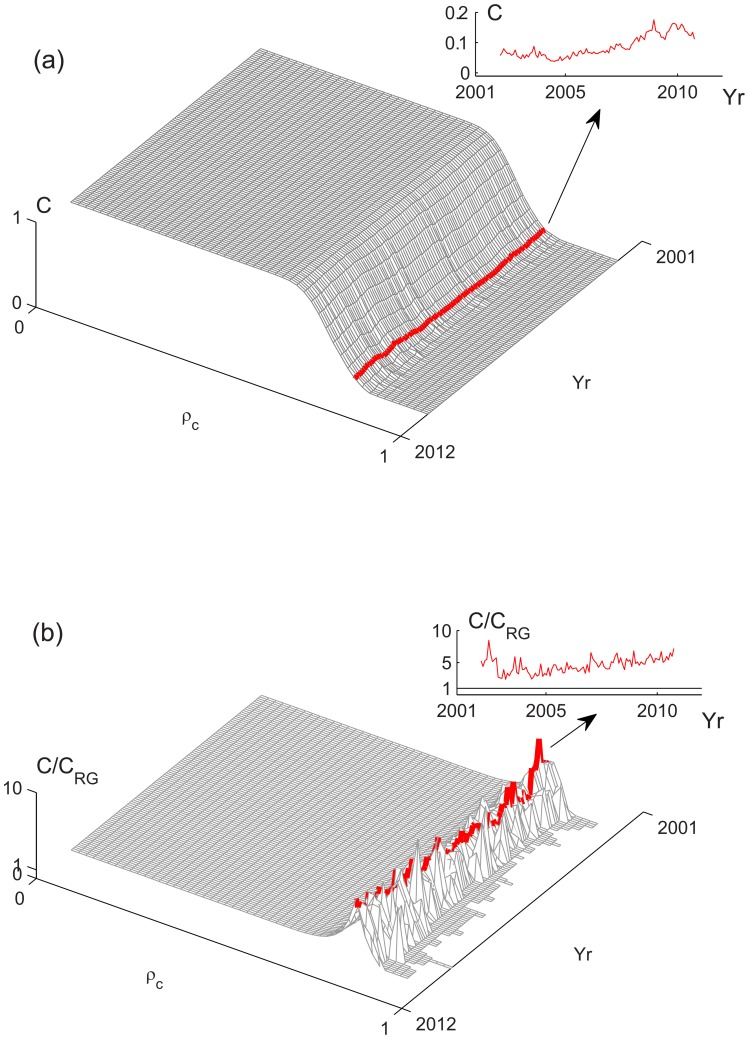
Network characterization of FIS, clustering coefficient of the network. (a) Clustering coefficient 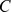 and (b) 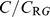 as functions of 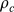 from 2001 to 2011. Typical results are shown for 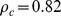. The corresponding degree PDF's at 2007 (“o”) and 2008 Yr (“+”) have been shown in FIG. 5a.

In light of the above, we take a network approach to further characterize the FIS in the closing price. Here, 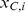, 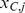 are viewed as nodes and considered “connected” if 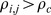 for a threshold value 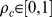. The *degree* of 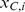 in the network is then defined in the usual way by counting its links, 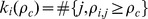 [23]–[26]. While no meaningful dynamics may be captured for 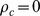 (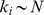) or 1 (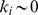), there exists an interesting 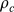 range, 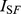, where a scale free configuration is revealed with a power law degree PDF 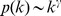 (dropping the subscript 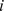) (FIG. 5a). The exponent 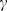 in the pre- and post-2008 regimes are averaged to 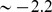 and 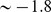, respectively (FIG. 5d), indicating more links are formed in the post-2008 regime and, possibly, more clustering in the network. The latter can be confirmed from the clustering coefficient 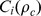, which calculates the ratio of the number of links among 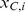
's neighbors versus a fully connected neighborhood. We used the average 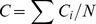 to describe this property of the network as a whole. FIG. 6 shows 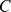 and 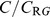 as the function of 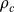 in the analyzing period, where 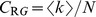 is the clustering coefficient of the random graph [23], [26]. For 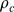 in 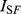, 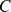 exhibits a similar rising trend as the FIS indicators through 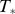 (FIG. 6a). It follows intuitively that the rising FIS indicators imply more price series showing similar fluctuation pattern, which in turn leads to more clustering among the 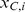
's. It is also seen a higher ratio 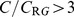 in the post-2008 regime, giving the evidence of a nontrivial networking structure underlying the market synchrony.

## Discussion and Conclusion

The notion of synchronized broadband processes in general should rest on the statistical ground of certain phase variable, and possibly be supported by the observation of similar fluctuation pattern. In this work, we adopt these premises to establish the preliminary ideas for synchrony in broad-band processes showing scale free characteristics. In particular, we propose using the wavelet transform to associate the phase to the large amplitude jump characterized as the singularity. We then suggest the state of FIS with the assumption of a common mechanism underlying the singular fluctuation. While these ideas are successfully demonstrated using artificial examples, we stress the importance of the “selectiveness” of the analyzing wavelet in wavelet transform. When it is too sensitive, many WTMMLs can emerge, which may be unrelated to the phase setting event leading to the large amplitude jump. We suggest using the wavelet that has the lowest number of vanishing moment, so as to associate the “most singular” fluctuation to the phase event.

The application of the proposed ideas to the market data leading to the 2008 financial crisis reveals several nontrivial results. The main implication of the findings is the significance of the market synchrony and its variability. The match of the rising FIS indicators 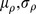 to the approach of the 2008 financial crisis gives promise to using FIS to capture significant market events. In particular, our results imply many price series showing similar fluctuation pattern is a troubling sign. Considering 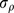 as a function of 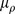 reveals a bifurcation pattern which suggests the transition towards a qualitatively different dynamical regimes in the crisis. It further implies that 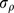, the FIS variability, may be considered generally as an order parameter of the overall market dynamics.

Lillo and collaborators also suggest synchronous market activities based on the persistent bid-ask spread in the market limit orders [27]–[29]. The spread is significant since it creates a supply-demand unbalance which can lead to large price fluctuation. On that ground, the association of the singular fluctuation of the price to an underlying mechanism is well supported. In this work, we offer two modest extensions: (a) in addition to an individual stock, market synchrony is a global property that generally exists between different stocks, and (b) there is a likely link between a market in turmoil and excess FIS, and FIS variability.

Finally, we suggest that similar price fluctuation in a group of stocks without obvious business link reflects the herding or collective behavior of the traders [31]–[33]. To this end, we observe similar FIS characteristics in the regular trading hours, suggesting traders' “collective motion” as a potential cause of the present findings. It is also in this realm that the market may be viewed as a “social” entity driven by a profit gaining objective. Indeed, market dynamics has been much discussed in such social context in the past; see [34], [35] and references therein. Along these lines, we remark that our attempt of using a network analogy to characterize the dynamics underlying FIS has yielded similar 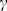 and 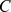 values as other social networks reported in the past [23]–[26]. In general, we believe FIS describes a genuine property in complex dynamics and should be explored to uncover subtle interaction among the coupled oscillators in large dynamical systems.

## Supporting Information

File S1 The ticker symbols for the price series
(DOCX)
