# Supplementary material for: Synchrony in Broadband Fluctuation and the 2008 Financial Crisis
Source: PLoS One. 2013 Oct 28;8(10):e77254. doi: 10.1371/journal.pone.0077254 (PMC3810396; doi:10.1371/journal.pone.0077254)
Supplement: File S1 — The ticker symbols for the price series (DOCX) [file pone.0077254.s001.docx]

The ticker symbols for the price series are: A, AA, ABC, ADI, ADM, AEE, AEP, AES, AET, AFL, AIV, AKS, ALL, AMD, AN, ANF, APD, APH, ARG, ATI, AVB, AVP, AVY, AZO, BBT, BBY, BCR, BDX, BF.B, BIG, BLK, BMS, BSX, BXP, CAG, CAH, CAM, CB, CCE, CCL, CEG, CHK, CI, CLF, CLX, CMA, CMI, CMS, CNP, CNX, COF, COG, COH, CPB, CSC, CVC, CVH, DF, DGX, DHI, DNB, DNR, DO, DOV, DRI, DTE, DV, DVA, ECL, ED, EFX, EIX, EL, EMN, EP, EQR, EQT, ETN, ETR, EW, FDO, FE, FHN, FII, FLR, FLS, FMC, FRX, FTR, GAS, GCI, GPC, GPS, GR, GT, GWW, HAR, HCN, HCP, HES, HIG, HNZ, HOG, HP, HRB, HRL, HRS, HST, HSY, HUM, IFF, IGT, IP, IPG, IR, IRM, ITT, IVZ, JBL, JCI, JCP, JEC, JNS, JWN, K, KEY, KIM, KMX, KR, KSS, L, LEG, LEN, LH, LLL, LM, LNC, LSI, LTD, LUK, LUV, LXK, M, MAR, MAS, MCK, MCO, MHP, MKC, MMC, MOS, MRO, MSI, MTB, MUR, MWV, NBL, NBR, NE, NFX, NI, NOC, NU, NUE, NWL, OI, OKE, OMC, PBI, PCG, PCL, PEG, PGN, PGR, PH, PHM, PKI, PLL, PNW, POM, PPG, PPL, PSA, PWR, PXD, R, RDC, RF, RHI, RHT, RL, ROK, ROP, RRC, RSG, RTN, S, SCG, SEE, SHW, SJM, SLE, SLM, SNA, SRE, STI, STJ, STT, STZ, SUN, SVU, SWK, SWN, SWY, SYK, SYY, TAP, TE, TEG, TER, THC, TIE, TIF, TMK, TMO, TSN, TSO, TSS, TXT, TYC, UNM, VAR, VFC, VLO, VMC, VNO, VTR, WAT, WDC, WEC, WFR, WHR, WMB, WPI, WPO, WY, X, XEL, XL, XRX.
